# A modified total arch replacement combined with a stented elephant trunk implantation for acute type A dissection under deep hypothermic circulatory arrest and selective antegrade cerebral perfusion

**DOI:** 10.1186/s13019-014-0140-6

**Published:** 2014-08-30

**Authors:** Su-Min Yang, Ping Xu, Cheng-Xiang Li, Qiang Huang, Hong-Bo Gao, Zhen-Fu Li, Qing Chang

**Affiliations:** Department of Cardiovascular Surgery, the Affiliated Hospital of Medical College, Qingdao University, Qingdao, 266003 Shandong China

**Keywords:** Dissection, Aortic arch surgery, Total arch replacement, Aortic surgery, Surgical therapy

## Abstract

**Objectives:**

Since the optimal management of patients with acute aortic dissection is unclear, this study analyzed total arch replacement combined with stented elephant trunk implantation in the treatment of acute type A aortic dissection.

**Methods:**

Between February 2008 and February 2013, 86 consecutive patients admitted to our hospital for acute type A dissection underwent total arch replacement combined with stented elephant trunk implantation under deep hypothermic circulatory arrest. The Bentall, David, and Wheat procedure was performed on 46, 12 and two patients, respectively. Ascending aorta replacement was performed on 26 patients, while two patients in Bentall group and 7 in ascending aorta replacement group underwent coronary artery bypass grafting as a concomitant procedure.

**Results:**

Sixty-nine patients were male and 17 patients were female, with an average age of 45.2 ± 2.3 years. The in-hospital mortality rate was 5.8%. Two patients presented with persisting paraplegia. The cardiopulmonary bypass time was 186.3 ± 45.2 minutes and the myocardium ischemia time was 102.6 ± 28.1 minutes. Selective antegrade cerebral perfusion time was 29.4 ± 10.3 minutes. Low-body circulatory arrest time was 18.5 ± 8.4 minutes. Mechanical ventilation time was 80.7 ± 11.3 hours. ICU and hospital stays were 5.3 ± 4.8 and 16.8 ± 5.5 days, respectively. Seven patients underwent reoperation for bleeding. During a mean follow-up of 28.5 months, two patients died and 2 patients were lost to follow-up. Obliteration of the false lumen around the stented graft and at the diaphragmatic level occurred in 97.1% (68 of 70) and 70% (49 of 70) of the patients.

**Conclusions:**

Modified total arch replacement combined with stented elephant trunk implantation using selective antegrade cerebral perfusion is a safe and effective alternative for patients with acute type A dissection and produces satisfactory clinical outcomes in our center.

**Electronic supplementary material:**

The online version of this article (doi:10.1186/s13019-014-0140-6) contains supplementary material, which is available to authorized users.

## Background

Acute type A dissection that conjointly involves the ascending aorta, aortic arch, and descending aorta remains a challenge for surgeons and results in high morbidity and mortality [1],[2]. Several surgical approaches have been introduced [3]-[8], but the optimal approach for patients with acute type A dissection involving the aortic arch is still open to discussion.

Recently in our institute, patients with acute type A dissection involving the aortic arch have undergone total replacement of the ascending aorta and aortic arch combined with transaortic stented elephant trunk implantation. Cardiopulmonary bypass (CPB) and selective antegrade cerebral perfusion (SACP) were achieved by cannulation of the right axillary artery, Deep hypothermic circulatory arrest (DHCA) was adopted. In this study, we report our experience with this procedure and evaluate the most efficacious management strategy for this lethal condition.

## Methods

### Patient demographics and characteristics

From February 2008 to February 2013, 86 consecutive patients underwent total arch replacement with a four-branched prosthetic graft and stented elephant trunk implantation in the descending aorta in our hospital. Sixty-nine patients were male and 17 patients were female, with an average age of 45.2 ± 2.3 years. Detailed patient preoperative characteristics are listed in Table 1. Patients with acute type A dissection underwent total arch replacement if there was (1) a primary tear in the transverse arch or the proximal descending aorta; (2) serious involvement of the arch vessels; and (3) Marfan syndrome [9]. The study protocol was approved by the Committee for the Protection of Human Subjects at the Affiliated Hospital of Medical College, Qingdao University. Informed consent was obtained from each patient involved in this study.

**Table 1:** **Preoperative characteristics**

Variables		Values (Mean ± SD) or Cases (n)
Age (years)		45.2 ± 2.3
Gender	Male	69
	Female	17
Diabetes		12
Hypertension		73
Chronic renal dysfunction		5
COPD (chronic obstructive pulmonary disease)		8
Cerebral vasculopathy		3
Acute myocardium ischemia		13
Acute renal dysfunction		21
Acute gastrointestinal ischemia		8
Lower extremity ischemia		31
Carotid artery affected		16
Acute pericardial temponade		13
Hemothorax		8
Marfan syndrom		24
Primary tear	Ascending aorta	39
	Arch	33
	Proximal descending aorta	6
	Multiple	8

### Surgical procedure

Anesthesia was induced and maintained according to accepted, standard procedures. Invasive Blood pressure was invasively monitored in both the upper and lower limbs. A jugular bulb catheter and near-infrared spectroscopy sensors were placed for assessing adequacy of brain perfusion. A transverse incision was routinely made under the right clavicle, and the right axillary artery was exposed. A median sternotomy was performed for all patients, and the incision was extended a few centimeters above the manubrium. An arterial cannula was inserted into the right axillary artery (Fem-Flex, 18 F; Edwards Lifesciences, Irvine, CA), to a depth of 2-3 cm. The right atrium was cannulated with a double-stage cannula, and the left ventricle was vented through the right superior pulmonary vein. Routinely, CPB and SACP were obtained using the right axillary artery. The artery line was ordinarily bifurcated; one line was used for the right axillary artery and the other for antegrade perfusion through one limb of the four-branch prosthetic graft. During cooling, the brachiocephalic arteries were isolated. The ascending aorta was clamped and proximal ascending aorta was longitudinally opened, and antegrade perfusion of cold-blood cardioplegic solution was directly infused into the coronary ostia. An aortic root procedure was performed where indicated. When the nasopharyngeal temperature reached 18 to 22°C, circulatory arrest was initiated. The patient was placed in the Trendelenburg position, and the head was packed in ice. Unilateral SACP was started through the right axillary artery.

**Figure 1:**
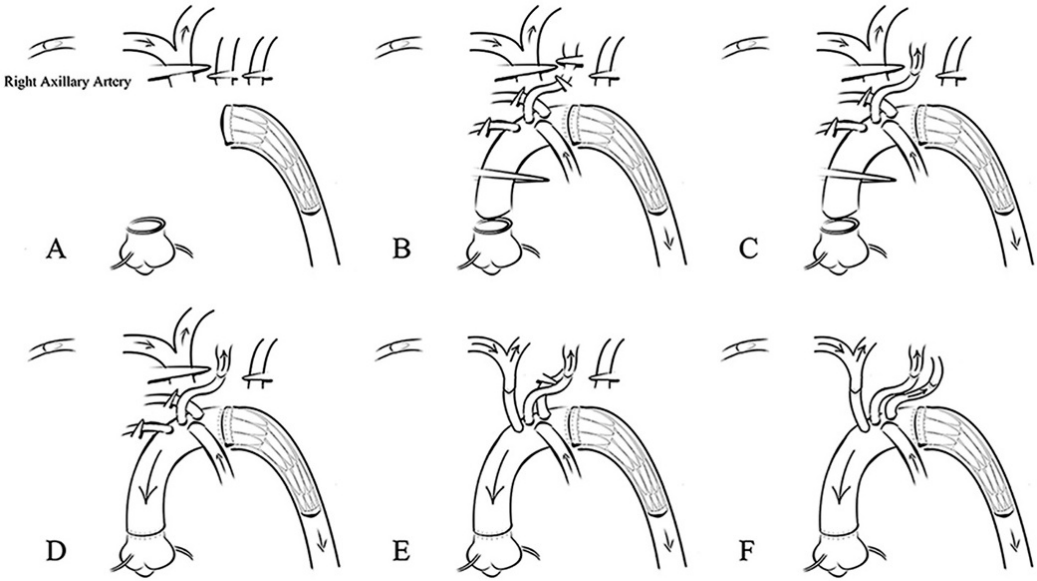
**Modified total arch replacement procedures. A** Skeletoned elephant trunk was implanted; **B** The distal end of the prosthetic graft was anastomosed to the transected aorta wall in a sandwich-like fashion and low-body was reperfused; **C** The left common carotid was anastomosed and reperfused; **D** The proximal end of the graft was anastomosed and the perfusion of myocardium was resumed: **E**-**F** The innominate and left subclavian arteries were anastomosed.

The arch was opened longitudinally, and the intimal tear was resected. Then, the arch was transected between the origins of the left common carotid and left subclavian arteries. We used the #26, #28 stented grafts (26 mm and 28 mm in diameter, respectively, and 120 mm in length. MicroPort Medical Co Ltd, shanghai, China). The size was determined by surgeons according to the preoperative CT. The stented graft is composed of self-expanded metal stent and graft. Before deployed, it was bundled. Once it was inserted into the aimed position, a pulling rod was pulled and the mental stent expand by itself (Figure 1A). The distal end of the prosthetic graft was firmly anastomosed to the transected aorta wall (adventitia and pruned intimal flap), incorporating the stented graft using the “open” aortic procedure without pledgets or Teflon-felts. Lower body reperfusion was initiated immediately following the aortic procedure by perfusing the limb of the four-branch prosthetic graft (Figure 1B) with half of the normal flow.

This modified strategy for total arch replacement was adopted by members of our surgical team. This sequence of anastomosis to the prosthetic graft was carried out from the left common carotid artery, proximal aortic stump, innominate artery, and left subclavian artery (Figure 1C-F). After the anastomosis to the left common carotid artery was accomplished, the brain was perfused bilaterally. CPB gradually resumed to its normal flow, and rewarming began.

## Results

### Surgical data

A total of 86 consecutive patients underwent total arch replacement and stented elephant trunk implantation. Detailed concomitant procedures are listed in Table 2. Detailed treatment parameters such as CPB and SACP time, circulatory arrest time, hospital and ICU stay, ventilation time, and unplanned revision are listed in Table 3.

**Table 2:** **Concomitant procedures**

	Bentall procedure	David procedure	Wheat procedure	Ascending aorta replacement	Coronary artery bypass grafting
Cases (n)	46	12	2	26	9

**Table 3:** **Intraoperative and postoperative data**

	Cases (n) or Values (Mean ± SD)	Reference
Unplanned revision for bleeding	7	
Permanent neurologic dysfunction	2	
CRRT	22	
Tracheotomy	5	
CPB (min)	186.3 ± 45.2	126---256
Myocardium ischemia (min)	102.6 ± 28.1	90---162
SACP (min)	29.4 ± 10.3	15---43
Low-body circulatory arrest (min)	18.5 ± 8.4	9---35
Nasopharyngeal temperature (°C)	20.2 ± 4.5	19---25
ICU stay (days)	5.3 ± 4.8	2---12
Mechanical ventilation (hours)	80.7 ± 11.3	19---156
Hospital stay (days)	16.8 ± 5.5	9---32
Cell saver transfusion (ml)	1385 ± 433.7	800---2200
Drainage on the first day (ml)	530 ± 142.8	320---1120
In-hospital mortality (%)	5.8	

CPB, cardiopulmonary bypass; CRRT, continuous renal replacement therapy; FFP, fresh frozen plasm; ICU, intensive care unit; RBC, red blood cells; SACP, selective antegrade cerebral perfusion.

### Morbidity and mortality

The in-hospital mortality rate was 5.8% (5 patients), and was mainly due to multiple organ failure. Five patients underwent tracheotomy because of hypoxemia and prolonged mechanical ventilation. Transient renal dysfunction presented in 22 patients who subsequently required continuous renal replacement therapy. Two patients were discharged with paraplegia. Seven patients had to undergo a second operation due to bleeding.

### Follow-up

Seventy-nine patients were followed-up for an average of 28.5 ± 7.8 months (from 3 to 55 months). Seventy patients underwent postoperative computed tomography. The prevalences of thrombosis of the false lumen around the stented graft and at the diaphragmatic level were 97.1% (68 patients) and 70% (49 patients). All patients presented with an enlargement of the true lumen and shrinkage in the diameter of the entire aorta. Two patients died for unknown reasons 6 and 15 months after surgery, and two patients with paraplegia were lost after hospital discharge. Injury to the spinal cord and organ ischmia was not observed during follow-up. All remaining patients survived without any new complications.

## Discussion

As one of the most catastrophic cardiovascular diseases, acute type A dissection has unfavorable clinical outcomes. Tsai et al. [1] reported that patients that undergo this procedure have an overall 32.5% in-hospital mortality rate and Weigang et al. [2] reported a 15.9% postoperative 30-day mortality rate in this patient population. Normally, emergency surgery is required and can significantly reduce mortality. In this study, we report our clinical experience with modified total arch replacement combined with stented elephant trunk implantation for acute type A aortic dissection.

Many procedures have been developed for the treatment of acute type A dissection. These include a conservative strategy with a simple replacement of the ascending aorta and hemiarch to a more aggressive one with a total aortic replacement. To improve late surgical outcomes, extended aortic replacement has been recommended for initial surgical treatment of acute type A dissection, and is not associated with higher morbidity and mortality [7],[10].

Spielvogel et al. [11],[12] have reported their experience with the use of a trifurcated graft and have shown that this procedure reduced the hypothermic circulatory arrest time to about 30 minutes. However, the graft used in the Spielvogel study was handmade, incorporating a different-diameter graft that increased the anastomotic stoma. It should be noted that most patients included in their study had an aneurysm and chronic dissection.

Since the introduction of the elephant trunk technique by Borst et al. [13] in 1983, several complications have been reported. Specifically, the soft graft located in the descending aorta was easy occluded and kinked, the recurrent laryngeal nerve was damaged, and it was very difficult to insert the soft graft into the true lumen [14]. In 1996, Kato et al. [3] described a partially stented graft, but this approach has also resulted in many complications that were reported in later studies [15]-[17]. Sun et al. [7] introduced their version of a modified elephant trunk procedure and showed that it had satisfactory clinical outcomes, making the Sun procedure (total arch replacement with stented elephant trunk implantation) a promising “standard” procedure for patients with acute type A dissection. Sun et al. [7] selectively perfused the brain via the right axillary artery, but the lower body was not perfused during DHCA. In their study, the rate of postoperative spinal cord injury was outstanding (2.1%), but they did not measure the low-body circulatory arrest time. Additionally, the strategy used for total arch replacement in the Sun et al. [7] study varied.

As a result of the problems stated above, we modified our surgical procedure using a four-branched graft concomitant with a stented elephant trunk, which handled better intraoperatively and produced medium term outcomes.

In terms of intraoperative handling, implantation of the stented elephant trunk in a bound and compressed state into the true lumen of a directly visible distal aorta is relatively straightforward. After the stented graft was implanted, the true lumen was expanded, substantiating the anastomosis to the distal end of prosthetic graft, thereby reducing the circulatory arrest time. The entire length of the elephant trunk was sustained by a stent, and the radial force exerted by the stented graft to the fragile aortic wall was uniform, avoiding the possible distortion of the prosthetic graft; in contrast to the segment of a prosthetic graft without a stent. The radial force was relatively low, minimizing the injury to the aortic wall. It is crucial that this procedure enlarges the true lumen, promotes thrombosis of the false lumen, and contributes to the shrinkage of the aorta. Thrombosis of the false lumen presented in most patients and enlargement of the true lumen and shrinkage of the entire aorta was seen in some patients.

Both CPB and SACP were performed using right axillary artery cannulation. Once the stented elephant trunk was successfully deployed and anastomosed with the distal end of the prosthetic graft, the perfusion of the lower body resumed. Song et al. [18] examined intermittent lower body perfusion with effective end-organ protection but they did not determine suitable flow and they performed the operative procedure under moderate hypothermia. In the ENREF_17 study, Luehr et al. [19] estimated a 120-minute ischemic tolerance of the spinal cord under deep hypothermia (20°C). The mean time of DHCA in our study was 18.5 ± 8.4 minutes, and the rate of spinal cord injury was 2.3% as a result of the administration of methylprednisolone during the surgical procedure, consistent with previous studies [7],[18]. Therefore, we believe that our DHCA strategy was safe. Moreover, the cannulation of the femoral artery may cause further damage and lower extremity complications. In our hospital, the femoral artery is used as an alternative approach in cases where the cannulation of the right axillary artery is difficult.

Since it was introduced in the late 20th century [20]-[22], profound hypothermia circulatory arrest and selective cerebral perfusion have provided favorable neuroprotection and clinical outcomes. As a result, they have been widely accepted as the standard strategy for transverse arch surgery. Clinicians are increasing body temperatures during this surgery to mitigate the systemic inflammatory response, avoid coagulation dysfunction and avoid the adverse effects associated with profound hypothermia and shortened CPB. The safest time for circulatory arrest of the spinal cord and end-organs (especially at a higher temperature) is still under debate. However, lower-body circulatory arrest is inevitably more dangerous when performed during a high body temperature. Leontyev et al. [23] recommended a more pronounced hypothermia, particularly when the operation was complex with a prolonged expected circulatory arrest time. Some investigators believe that profound hypothermia causes coagulation dysfunction and thereby, serious postoperative bleeding. However, the danger is possibly overestimated. According to a report by Milewski et al. [24], the rate of revision for bleeding was not higher in patients with profound hypothermia (3.8%) compared to those that had been placed in deep hypothermia (4.3%). However, Svensson et al. [25] reported a higher risk of coagulation dysfunction and end-organ failure associated with lower-body circulatory arrest under a higher temperature.

In this study, we modified the more common sequence of brachiocephalic artery anastomosis to the prosthetic graft such that anastomosis began with the left common carotid artery, and moved to the proximal aortic stump, innominate artery, and left subclavian artery. The SACP ceased when anastomosis to the left common carotid artery was completed, the flow was gradually returned to normal and rewarming began. Once anastomosis of the proximal aortic stump was complete, the myocardium was reperfused. In the present study, the duration of CPB and myocardium ischemia was relatively shortened: 186.3 ± 45.2 and 102.6 ± 28.1 minutes, respectively.

Despite our important observations, limited conclusions can be drawn from the present study because it was an observational and retrospective study and other surgical methods were not compared. The outcomes only represent the experience of a few skilled surgeons in a single center.

## Conclusions

In this report, we describe a modified anastomotic procedure in which the total arch was replaced with a four-branched prosthetic graft combined with an implanted stented elephant trunk under deep hypothermic circulatory arrest and selective antegrade cerebral perfusion. In our center, this modified procedure produced satisfactory clinical outcomes for the patients with acute type A dissection and was recommended as a safe and effective alternative treatment.

## Authors’ original submitted files for images

Below are the links to the authors’ original submitted files for images. Authors’ original file for figure 1

## Abbreviations

ICU: Intensive care unit
CPB: Cardiopulmonary bypass
SACP: Selective antegrade cerebral perfusion
DHCA: Deep hypothermic circulatory arrest
