# Supplementary figures and images for: A modified total arch replacement combined with a stented elephant trunk implantation for acute type A dissection under deep hypothermic circulatory arrest and selective antegrade cerebral perfusion
Source: J Cardiothorac Surg. 2014 Aug 30;9:140. doi: 10.1186/s13019-014-0140-6 (PMC4203861; doi:10.1186/s13019-014-0140-6)

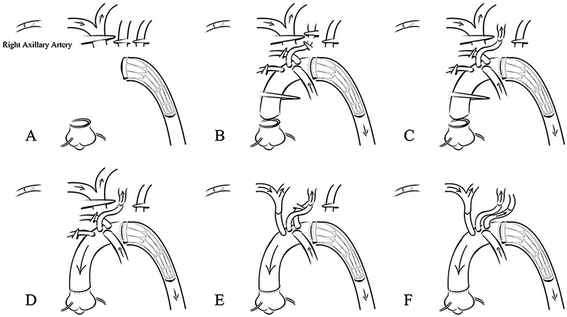

Supplement: Supplementary file 1 — Authors’ original file for figure 1 [file 13019_2014_140_MOESM1_ESM.gif]
